# Emergent Low-Frequency Activity in Cortico-Cerebellar Networks with Motor Skill Learning

**DOI:** 10.1523/ENEURO.0011-23.2023

**Published:** 2023-02-17

**Authors:** Pierson Fleischer, Aamir Abbasi, Andrew W. Fealy, Nathan P. Danielsen, Ramneet Sandhu, Philip R. Raj, Tanuj Gulati

**Affiliations:** 1 Center for Neural Science and Medicine, Department of Biomedical Sciences, Cedars-Sinai Medical Center, Los Angeles, California 90048; 2 Department of Neurology, Cedars-Sinai Medical Center, Los Angeles, California 90048; 3 Department of Medicine, David Geffen School of Medicine, University of California, Los Angeles, Los Angeles, California 90095; 4 Department of Bioengineering, Henry Samueli School of Engineering, University of California-Los Angeles, Los Angeles, California 92697

**Keywords:** cerebellum, motor cortex, oscillations, skill learning

## Abstract

The motor cortex controls skilled arm movement by recruiting a variety of targets in the nervous system, and it is important to understand the emergent activity in these regions as refinement of a motor skill occurs. One fundamental projection of the motor cortex (M1) is to the cerebellum. However, the emergent activity in the motor cortex and the cerebellum that appears as a dexterous motor skill is consolidated is incompletely understood. Here, we report on low-frequency oscillatory (LFO) activity that emerges in cortico-cerebellar networks with learning the reach-to-grasp motor skill. We chronically recorded the motor and the cerebellar cortices in rats, which revealed the emergence of coordinated movement-related activity in the local-field potentials as the reaching skill consolidated. Interestingly, we found this emergent activity only in the rats that gained expertise in the task. We found that the local and cross-area spiking activity was coordinated with LFOs in proficient rats. Finally, we also found that these neural dynamics were more prominently expressed during accurate behavior in the M1. This work furthers our understanding on emergent dynamics in the cortico-cerebellar loop that underlie learning and execution of precise skilled movement.

## Significance Statement

Movement execution involves parallel processing across brain regions, with the motor cortex (M1) being a key hub that recruits several subcortical nodes. The cerebellar cortex is a principal receiver of M1 projections via pons, but the emergent dynamics in these regions with motor skill learning is incompletely understood. We performed simultaneous recordings of M1 and cerebellum in a reach-to-grasp task. We found low-frequency activity and coordinated neural dynamics emerged within and across regions with skillful task execution. Recent interest in modulating cortico-cerebellar networks for motor recovery postinjury/stroke make this work an important precursor to assessing whether similar low-frequency activity in cortico-cerebellar networks can serve as a biomarker of motor recovery and help to optimize the modulation of these networks.

## Introduction

The primary motor cortex (M1) is viewed as a driver for movement, and an emerging view posits transient oscillatory dynamics—both at the level of spiking and local field potentials (LFPs)—as the neural substrate for it (Donoghue et al., 1998; Churchland et al., 2010, 2012; Mollazadeh et al., 2011; Hall et al., 2014a; Ramanathan et al., 2018; Lemke et al., 2019). There has been a particular interest in low-frequency quasi-oscillatory activity (LFOs) in M1, which can be brief (1–2 cycles) for rapid movements or longer for sustained movements, and it has been shown to be phase locked to submovement timing (Stefanics et al., 2010; Hall et al., 2014b; Ramanathan et al., 2018; Lemke et al., 2019). Recent work showed that such oscillatory dynamics are coordinated in the M1 and dorsolateral striatum in rodents as they learned a reach-to-grasp task (Lemke et al., 2019). One of the principal projections of M1 is to the cerebellum via the pons (Kelly and Strick, 2003; Leergaard et al., 2004; Wagner et al., 2019; Guo et al., 2021), but similar oscillatory dynamics have not been studied in cortico-cerebellar networks.

M1 is a key brain hub involved in voluntary forelimb movement: experimental lesions of M1 in animal models or neurologic injury to M1 (e.g., stroke) impair dexterity (Lawrence and Kuypers, 1968; Whishaw et al., 1993; Krakauer and Carmichael, 2017; Ramanathan et al., 2018); stimulation of M1 neurons evokes movement (Grünbaum and Sherrington, 1902; Graziano et al., 2002; Miri et al., 2017), spiking activity in M1 is closely linked to movement parameters (Evarts, 1966; Georgopoulos et al., 1982; Scott, 2003; Guo et al., 2015; Lemke et al., 2019; Wagner et al., 2019), and optogenetic perturbation of M1 affects forelimb behaviors (Guo et al., 2015; Miri et al., 2017; Bollu et al., 2018; Sauerbrei et al., 2020). The role of the cerebellum in the coordination of arm movements has also been extensively studied. Investigation of prehension/reaching tasks in animals have shown that cerebellar neurons—both in the cerebellar cortex and its deep nuclei—are tuned to several movement-related events, such as movement onset (MO); cues leading to movement; and its duration, limb position, velocity, and muscle activity (Thach, 1970; Wetts et al., 1985; Marple-Horvat and Stein, 1987; Fortier et al., 1989; van Kan et al., 1993; Heck et al., 2002). In addition to coding for the above-listed features of limbs and associated movement parameters, other evidence indicates that the cerebellum participates in the formation of procedural memories, learning, and retention of skills, habits, and conditioned responses (Bloedel et al., 1997; Thach, 1998). Cerebellar lesions impair acquisition of skilled behaviors and patients with cerebellar disease show impaired reaching (Zackowski et al., 2002; Tseng et al., 2007; Nashef et al., 2019). Furthermore, optogenetic perturbation of cerebellar nuclei or pontine inputs can cause a loss of end point precision in mice during reach-to-grasp behavior (Becker and Person, 2019; Guo et al., 2021). Additionally, electric stimulation over the cerebellum facilitates adaptive control of reaching (Galea et al., 2011; Herzfeld et al., 2014). Recent rodent work using two-photon imaging showed the emergence of shared neuronal dynamics in M1–cerebellar ensembles as animals learned to expertly control a manipulandum (Wagner et al., 2019).

In this study, we have focused on transient oscillatory dynamics that emerge in M1 and the cerebellum as a reaching skill is learned with contiguous 5 d of practice. We recorded neural activity in the M1 and contralateral cerebellum (the primary M1 target through pons nuclei) throughout the learning of a reach-to-grasp skill in rats. We observed emergent coordinated low-frequency oscillatory activity (1–4 Hz) across M1 and cerebellum LFPs that were linked to increased success rates of at least 30% by day 5. We also found that LFPs modulated spiking in both regions and that the spiking dynamics were conserved for successful, accurate movements.

## Materials and Methods

### Animal model and surgical procedures

**Figure 1.**
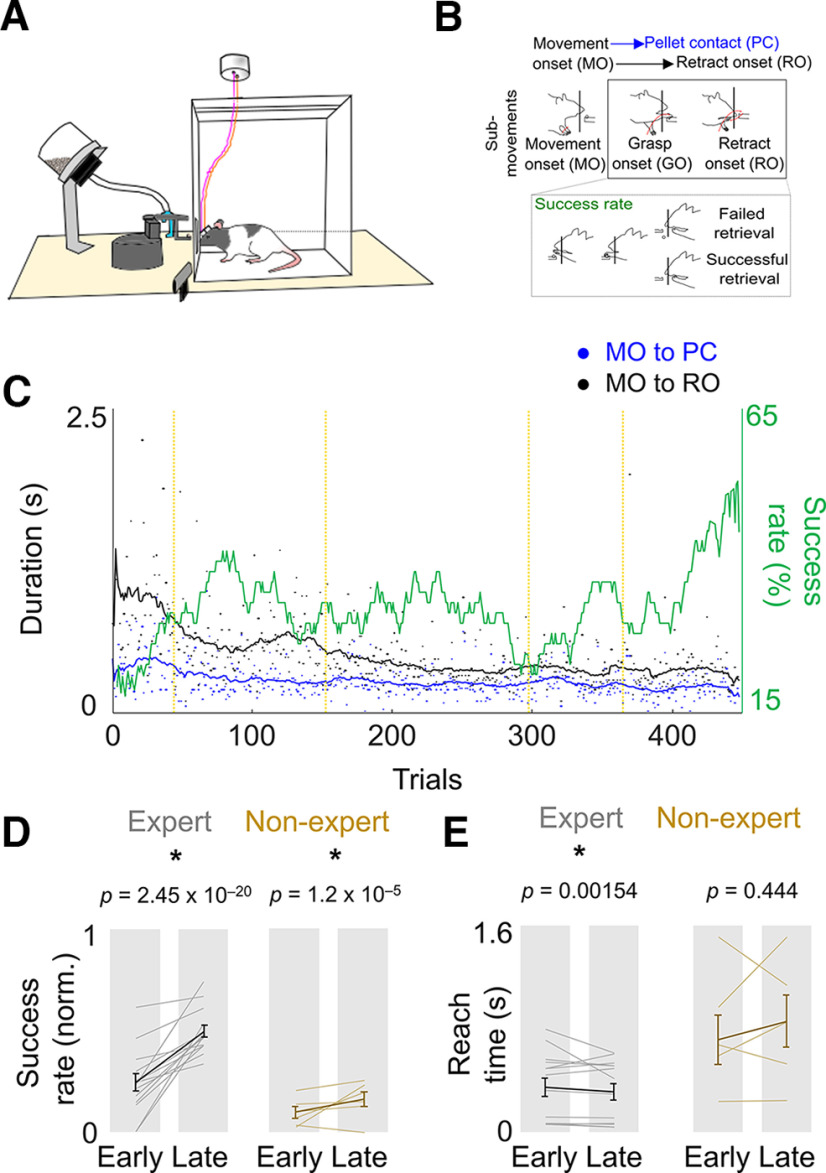
Behavioral evaluation of the skilled reach-to-grasp task. ***A***, The behavioral setup for the skilled forelimb reaching task with simultaneous neurophysiological recording. ***B***, Top, Illustration of the reach-to-grasp task showing the three major parts of the reach movement: reach onset, pellet contact, and retract onset. Bottom, Comparison of a failed trial and a successful trial. ***C***, Success rate and reach event timing from a representative expert animal. ***D***, ***E***, Difference in success rate and reach duration from early training days to late training days [*n* = 14 experts (left, gray lines), 6 nonexperts (right, mustard lines)]. Thin lines represent individual animals, and the thick line is the mean and SEM across animals. The *p*-values are from mixed-effects models.

**Figure 2.**
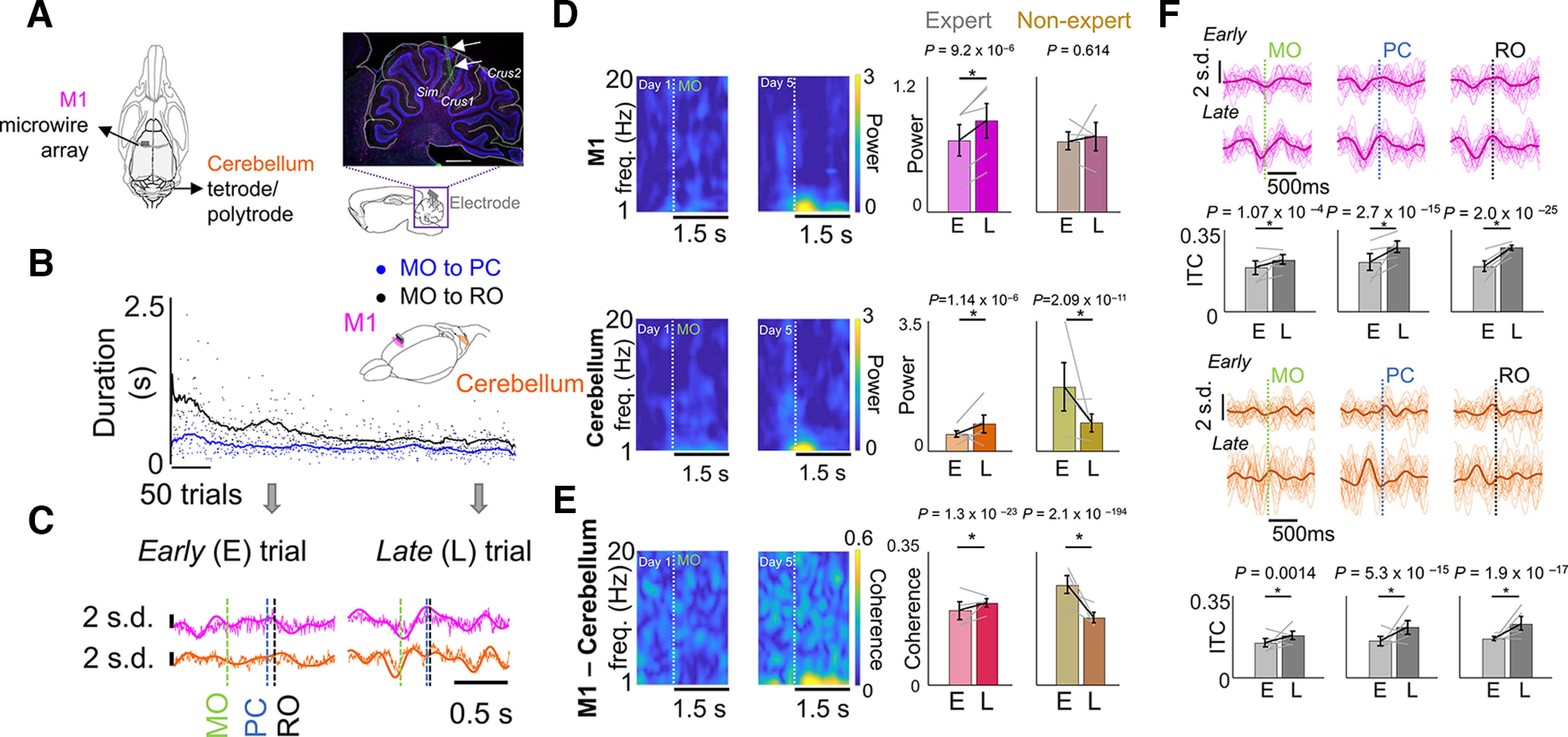
Coordinated movement-related mesoscopic activity emerges across M1 and cerebellum during skill learning. ***A***, Left, Schematic of recording electrode locations in M1 and contralateral cerebellum depicted from top. Right, Histologic verification of recording location in cerebellum [three markers used: Iba1 (green, microglia), GFAP (pink, astrocytes), DAPI (blue, nuclei)]. Sagittal section shows cerebellar lobules and cortical layers. Scale bar, 1 mm. Electrode shank is marked by two arrows. ***B***, Example time course of skill learning from an expert animal and illustration of recording scheme in M1 and the cerebellum from a frontal-side view (MO, movement onset; PC, pellet contact; RO, retract onset). ***C***, M1 (pink) and cerebellum (orange) LFP for representative successful trials from days 1 (early) and 5 (late) trials in an expert animal; 1–4 Hz filtered LFP is overlaid on raw trace. ***D***, Left, Spectrograms of example M1 and cerebellar channels from an expert animal. Right, Difference in 1–4 Hz cerebellum and M1 power from early training to late training in experts and nonexperts. The gray lines represent the mean power from individual expert and nonexpert animals [left bar plot (experts), *n *=* *5 animals; right bar plot (nonexperts), *n *=* *4 animals], and the black lines represent the mean ± SEM. The *p*-values are from mixed-effects models. ***E***, Left, Coherograms from an example M1 and cerebellum LFP channel pair. Right, Difference in 1–4 Hz M1–cerebellum coherence from early to late training sessions in experts and nonexperts. The gray lines represent the mean coherence from individual animals (*n *=* *4 animals each in expert and nonexpert bar plots), and the black lines represent the mean and SEM. The *p*-values are from mixed-effects models. ***F***, The 1–4 Hz filtered LFP from example M1 and cerebellum channels time locked to reach events; individual trials with mean overlaid. Bar plots depict the changes in ITC from early trials to late trials (top row, M1; bottom row, cerebellum). The gray lines represent the mean ITC from individual animals (*n *=* *5 animals), and the black lines represent the mean and SEM; *p*-values are from mixed-effects models.

**Figure 3.**
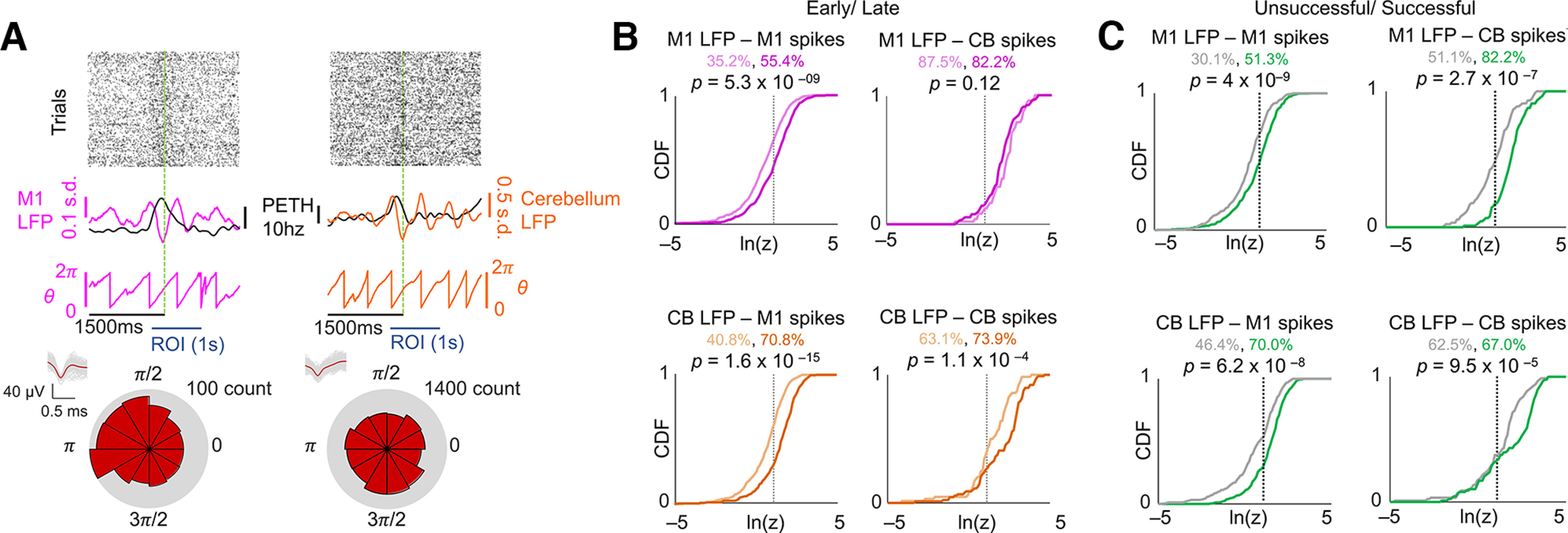
Coordinated spiking activity emerges across M1 and cerebellum during skill learning. ***A***, Illustration of spike locking to LFP phase for M1 unit–M1 LFP (left) and cerebellum unit–cerebellum LFP (right) pair examples. Top, Raster plots of reach-centered spiking activity from example single units. Middle, the 1–4 Hz filtered LFP overlayed with PETHs from example unit. Below is the extracted phase from the filtered LFP. Bottom, Polar histograms of the spikes that occurred at various LFP phases. ***B***, Cumulative density functions (CDFs) of the *z*-statistic for every LFP–unit pair across and within each region. The vertical dotted lines indicate the significance threshold (*p *=* *0.05). The percentage of the pairs with significant *p*-values is displayed. Lighter colors indicate early trials, and darker colors indicate later trials. *n *=* *428 M1 unit–LFP pairs on day 1; *n *=* *358 M1 unit–LFP pairs on day 5; *n *=* *66 cerebellum unit–LFP pairs on day 1; *n *=* *103 cerebellum unit–LFP pairs on day 5. The *p*-values derived using a Kolmogorov–Smirnov test. ***C***, Success/failure CDFs of the *z*-statistic for every LFP–unit pair within and across each region on day 5. The vertical dotted lines indicate the significance threshold (*p *=* *0.05). The percentage of the pairs with significant *p*-values is displayed. Green indicates successful trials, and gray indicates failures. The *p*-values were derived using a Kolmogorov–Smirnov test.

**Figure 4.**
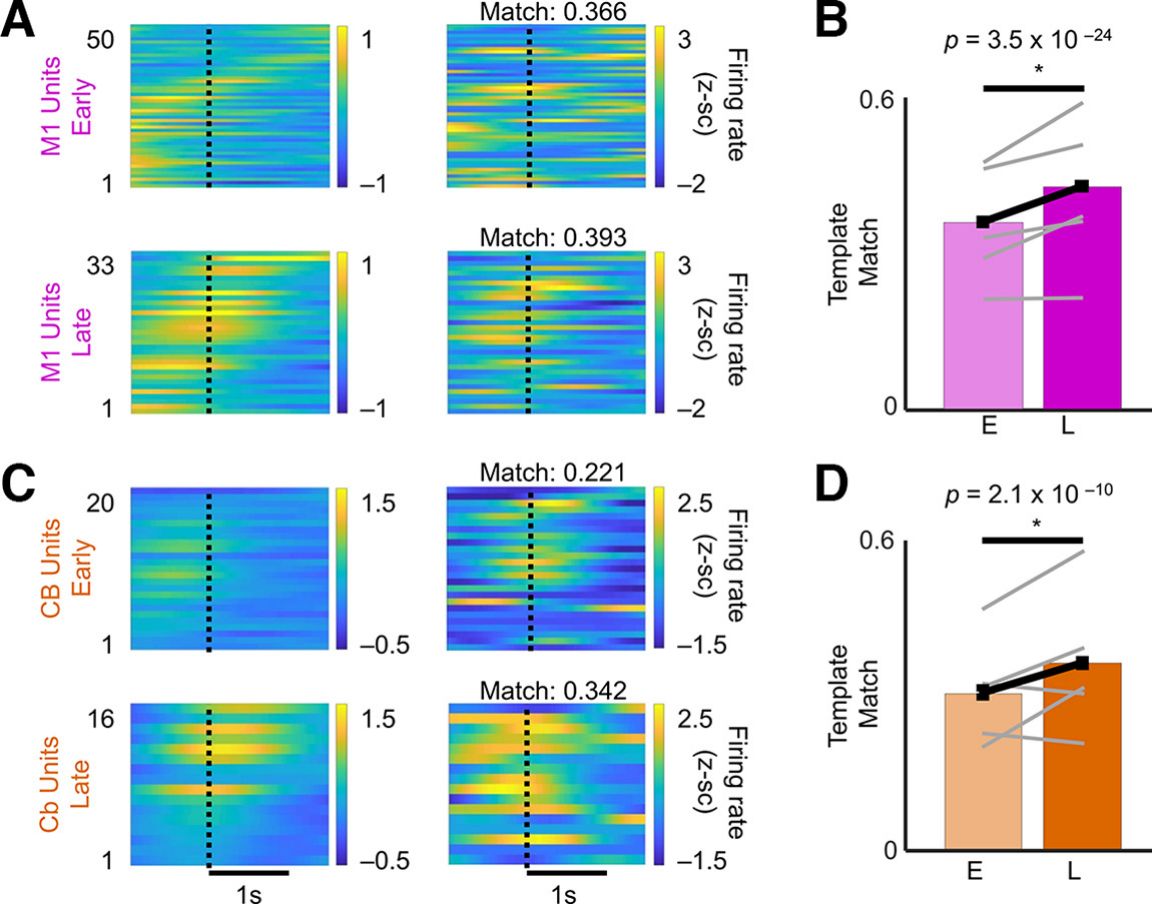
Changes in M1 and cerebellum neural dynamics with skill learning. ***A***, M1 successful trial averaged the PETHs from an example rat (left) and a single-trial PETH example (right) for early (top) and late (bottom) training sessions. ***B***, M1 PETH template match over training. Bars indicate mean ± SEM over trials. Gray lines indicate average per animal (*n *=* *5 animals). The *p*-values are from mixed-effects models. ***C***, Cerebellum successful trial-averaged PETHs from an example rat (left) and single-trial PETH example (right) for early (top) and late (bottom) training sessions (CB: cerebellum). ***D***, Cerebellum PETH template match over training. Bars indicate the mean ± SEM over trials. Gray lines indicate the average per animal (*n *=* *5 animals).

**Figure 5.**
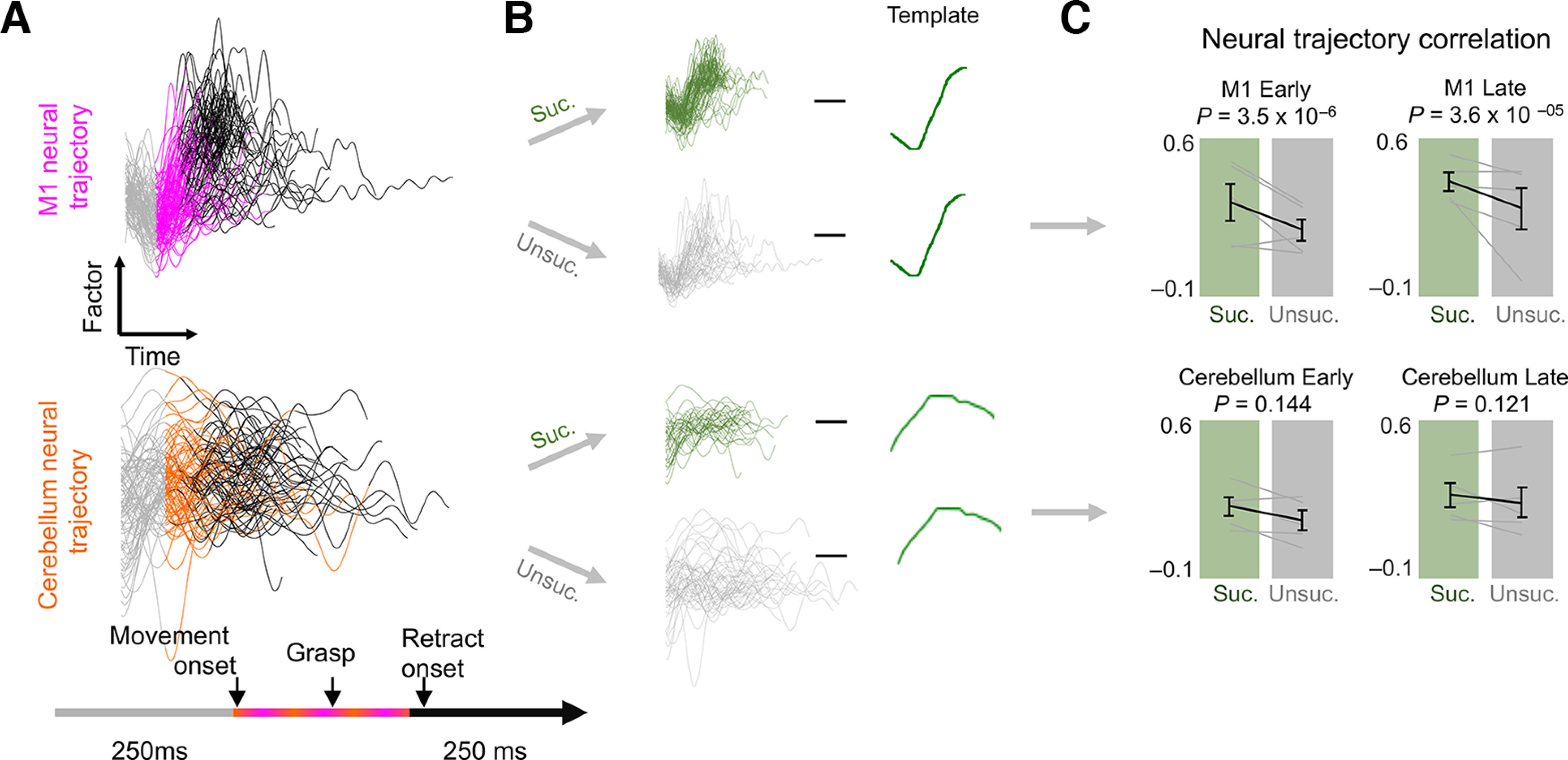
Skilled movement representation in M1 and cerebellum. ***A***, Example GPFA neural trajectories from late training sessions for M1 (top) and cerebellum (bottom) in a single animal. ***B***, Illustration of the process of comparing factor trajectories from successful and unsuccessful trials to the template (mean successful trajectory). ***C***, Deviation from the template for M1 (top) and cerebellum (bottom) factors. Gray lines represent individual animals (*n *=* *5 animals), and the black line is the mean ± SEM across animals. The *p*-values are from mixed-effects models.

All procedures were conducted in accordance with protocols approved by the Institutional Animal Care and Use Committee at the Cedars-Sinai Medical Center. Adult male Long Evans rats (*n* = 20; weight, 250–400 g; Charles River Laboratories) were housed in a 14 h/10 h light/dark cycle. All experiments were performed during the light cycle. We used 10 rats for behavior only (Fig. 1) and 10 rats for behavior and physiology (Figs. 2-5, Table 1, details). No statistical methods were used to predetermine cohort size, but our sample sizes are similar to those reported in previous publications (Kargo and Nitz, 2004; Ramanathan et al., 2015; Sauerbrei et al., 2015; Gulati et al., 2017; Lemke et al., 2019). Animals were pair-housed before electrode implantation or behavioral training and then single-housed after to prevent damage to implants or to implement food restriction, respectively.

**Table 1:** Number of rats used for experiments

Animal	Camera framerate	M1 Probe	M1 units	Cb Probe	Cb units
1	30	32-Channel array	208	4 × 8 Tetrode	117
2	75	32-Channel array	737	4 × 16 Polytrode	236
3	75	32-Channel array	482	4 × 16 Polytrode	51
4	75	32-Channel array	870	4 × 16 Polytrode	Discarded
5	87	Not implanted	N/A	2 × 32 Polytrode	150
6	303	32-Channel array	533	4 × 16 Polytrode	123
7	30	No	N/A	No	N/A
8	30	No	N/A	No	N/A
9	30	No	N/A	No	N/A
10	30	No	N/A	No	N/A
11	30	No	N/A	No	N/A
12	30	No	N/A	No	N/A
13	87	No	N/A	No	N/A
14	87	No	N/A	No	N/A
**15**	**30**	**32-Channel array**	**N/A**	**4 × 8 Tetrode**	**N/A**
**16**	**30**	**32-Channel array**	**N/A**	**4 × 8 Tetrode**	**N/A**
**17**	**75**	**32-Channel array**	**N/A**	**4 × 16 Polytrode**	**N/A**
**18**	**75**	**32-Channel array**	**N/A**	**4 × 16 Polytrode**	**N/A**
**19**	**87**	**No**	**N/A**	**No**	**N/A**
**20**	**87**	**No**	**N/A**	**No**	**N/A**

Tabulated list of animals and behavioral monitoring camera frame rates and electrode used (see columns). Rows in bold type indicate animals that did not gain expertise in the task (spike data were not analyzed in these animals). N/A, Not applicable.

All surgical procedures were performed using sterile techniques under 1–4% isoflurane. Surgery involved cleaning and exposure of the skull and preparation of the skull surface using cyanoacrylate and then implantation of the skull screws for referencing and overall head-stage stability. The analgesic regimen included the administration of 0.1 mg/kg body weight buprenorphine, and 5 mg/kg body weight carprofen. Neural implanted rats were also administered 2 mg/kg body weight dexamethasone and 33 mg/kg body weight sulfatrim for 5 d. All neural-implanted animals were allowed to recover for 5 d before further behavioral training. Ground and reference screws were implanted posterior to λ contralateral to the recorded cerebellum, contralateral to the neural recordings. For M1 recordings, 32-channel arrays (33 μm polyamide-coated tungsten microwire arrays) were lowered to a depth of ∼1200–1500 μm in either the left or right M1 depending on handedness. These were implanted centered at 0.5 mm anterior and 3 mm lateral to the bregma (Ramanathan et al., 2015; Lemke et al., 2019). For cerebellar recordings, we used 32–64 channel tetrodes (NeuroNexus) or shuttle-mounted polytrodes (Cambridge NeuroTech). The probes were lowered into the cerebellar cortex through a craniotomy centered at 12.5 mm posterior and 2.5–3 mm lateral to bregma. Shuttle mounted probes were moved across days and recorded from depths of 1.5–4 mm. Our target regions were Simplex/Crus I and Crus II areas of the cerebellum. Activity in these areas has shown modulation during upper limb motor behaviors and in response to corticofugal fiber and forelimb stimulation (Atkins and Apps, 1997; Baker et al., 2001; Heck et al., 2007). For the cerebellar recordings, we confirmed the location of electrode tips either through the following: (1) staining with the orange/red fluorescence stain DiI (Thermo Fisher Scientific); or (2) three markers of Iba1 (microglia), GFAP (astrocytes), DAPI (nuclei), as shown in Figure 2*A* (also see immunohistochemistry details below).

### Experimental design

Rats were acclimated to the behavioral box for at least 2 d and then exposed to a reach-to-grasp task for 5–10 trials to establish hand preference before neural probe implantation. Probe implantation was performed in the contralateral M1 and ipsilateral cerebellum to the preferred hand. Rats were allowed to recover for at least 5 d before the start of experimental sessions. During behavioral assessments, we monitored the animals and ensured that their body weights did not drop to <90% of their initial weight. We used an automated reach-box, controlled by custom MATLAB scripts and an Arduino microcontroller. This setup requires minimal user intervention, as described previously (Wong et al., 2015). Each trial consisted of a pellet dispensed on the pellet tray, followed by an alerting beep indicating that the trial was beginning. They then had 15 s to reach their arms through the slot, and grasp and retrieve the pellet. A real-time “pellet detector” using an infrared sensor centered over the pellet was used to determine when the pellet was moved, which indicated that the trial was over, and the door was closed. All trials were captured by video through a camera placed on the side of the behavioral box. The camera was synced with the electrophysiology data using Arduino digital output or directly through TTL (transistor–transistor logic) pulses to the TDT RZ2 system (Tucker-Davis Technologies). In electrode-implanted animals, the video frame rate ranged from 30 to 303 Hz (Table 1). For behavior-only animals, the frame rate was either 30 or 87 Hz.

### Behavioral testing

Rats began behavioral testing training 5 d after surgery by performing the same reach-to-grasp task. Electrophysiology recordings were taken throughout the full extent of the testing, which consisted of one to two sessions of 60–100 trials/d for 5 d. Typically, each day would consist of a session of 100 trials followed by a session of 60 trials. Sessions within a day were spaced by a 2 h resting block.

#### *In vivo* electrophysiology

Units and LFP activity were recorded using a 128-channel TDT-RZ2 system (Tucker-Davis Technologies). Spike data were sampled at 24,414 Hz, and LFP data were sampled at 1017.3 Hz. ZIF (zero insertion force) clip-based digital head stages from Tucker-Davis Technologies were used that interface the ZIF connector and the Intan RHD2000 chip that uses 192× gain. Behavior-related time stamps (i.e., trial onset, trial completion) and video time stamps (i.e., frame times) were sent to the RZ2 analog input channel using an Arduino digital board and synchronized to the neural data.

#### Immunohistochemistry

After all experiments, rats were anesthetized and transcardially perfused with 1% PBS, followed by 4% paraformaldehyde (PFA). The harvested brains were postfixed for 72 h in PFA and immersed in 30% sucrose. For immunofluorescence staining (Fig. 2*A*), sagittal cerebellar tissue cryostat sections (40 μm) were washed 3× in 1× Tris-buffered saline (TBS), followed by antigen retrieval with 0.1N hydrochloric acid (HCl). After three more washes in 1× TBS, sections were blocked with 5% normal donkey serum in 0.1% TBS-T (Triton X-100) for 1 h. Sections were then incubated in primary antibodies for astrocytes and microglia overnight. The next day, sections were washed three times in 1× TBS and then incubated with fluorescent secondary antibodies for 2 h. Sections were then washed three times in 1× TBS and incubated with 300 nm DAPI in 1× TBS for 7 min, before coverslipping with mounting media (ProLong Glass Antifade Mountant; catalog #P36980, Thermo Fisher Scientific). Primary antibodies used are 1:1000 rat-anti-GFAP (catalog #13–0300, Thermo Fisher Scientific) and 1:1000 rabbit-anti-IBA1 (catalog #019–19 741, Wako). Secondary antibodies used are 1:250 Alexa Fluor-647 donkey-anti rat (catalog #712–605-153, The Jackson Laboratory) and 1:1000 Alexa Fluor-488 donkey-anti rabbit 488 (catalog #A-21206, Thermo Fisher Scientific). Fluorescent sections were imaged with a BZ-X700 microscope (Keyence).

### Analyses details

Analyses were conducted using custom-written scripts and functions in MATLAB 2018a.

#### Behavioral analysis

Behavioral analysis was performed based on video recorded during experimental sessions. Reach videos were viewed and manually scored to obtain trial success, hand position, and time points for reach onset, pellet contact (PC), and retract onset (RO). To characterize motor performance, we quantified pellet retrieval success rate (percentage of pellets successfully retrieved into the box) and reach duration (time from reach onset to retract onset). We classified animals as expert and nonexpert based on a success rate of at least 30% by day 5. Based on this classification, we found that 14 animals were experts and 6 animals did not achieve expertise in the task in 5 d of practice.

#### Local field potential analyses

Artifact rejection was first performed on LFP signals to remove broken channels and noisy trials. LFPs were then *z*-scored and median referenced separately for M1 and cerebellum. One of the nine implanted animals had excessive noise in the cerebellar recordings, and hence cerebellar spike and LFP data were not used from this animal (Table 1, Animal 4). To compensate for this, one animal with only cerebellum implants was included in the cohort (Table 1, Animal 5). LFP power was calculated on a trial-by-trial basis and then averaged across channels and animals, with wavelet decomposition using the EEGLAB (Delorme and Makeig, 2004) function “newtimef.” To characterize the coordination of activity across regions, we measured changes in movement-related spectral coherence between LFP channels in M1 and cerebellum. For learning comparisons, coherence was measured for the same channels on early days (E; days 1 and 2) and late days (L; days 4 and 5), and specifically for channels with an increase in power of 0.5 baseline-normalized unit from early to late days. Strong coherence in a specific frequency band indicates a constant phase relationship in that frequency between two signals and is theorized to indicate increased communication between regions (Fries, 2005, 2015; Lemke et al., 2019). M1–cerebellum LFP coherence was calculated for each pair of channels using the EEGLAB function “newcrossf” with 0.1 s windows moving by 0.01 s.

To determine whether the emergence of coordinated low-frequency activity during training was attributable solely to faster movements, we compared LFP power and coherence between “fast” trials (trials with a movement duration <300 ms) on days 1 and 2 versus days 4 and 5.

For analyses in Figures 2 and 3, we filtered the LFP signals to isolate and display the low-frequency (1–4 Hz) component of the signal. Filtering was performed using the EEGLAB function “eegfilt” (Delorme and Makeig, 2004). In addition to display purposes, we also used filtered LFP to characterize the phase locking of spiking activity specifically to low-frequency LFP signals. For this, we used the Hilbert transform linear operator (MATLAB) to extract the phase information from low- frequency filtered LFP signals (Fig. 3).

To quantify the phase locking of LFP signals to specific submovements (MO, PC, and RO), we calculated the inter-trial coherence (ITC) of LFP signals across trials over a 1 s window centered on each submovement (Fig. 2*C*). ITC was measured and compared for the same channels on early and late days across all channels (except those removed because of noise). ITC was computed using the EEGLAB function newtimef (Delorme and Makeig, 2004).

#### Spiking analyses

Automated spike sorting was performed using Spyking Circus (Yger et al., 2018). High-pass filtering and local-median subtraction was performed on the raw data to obtain spike data. Spikes are detected as threshold crossings, and extracellular waveforms and spike times were isolated. Spikes were projected into a lower-dimensional feature space using principal component analysis. Then, clustering and template matching was done to isolate putative spike times and waveforms from individual neurons. Finally, manual curation was performed to identify well isolated units that are selected for this analysis. All units were analyzed without defining their cell type based on waveform shape. Behavior-related time stamps (trial onset and trial completion) were sent to the RZ2 analog input channel using an Arduino digital board and synchronized to neural data.

#### Unit modulation and spike–LFP phase analysis

Spikes were binned at 25 ms and time locked to behavioral markers. For visualization purposes, the perievent time histogram (PETH) was estimated by the MATLAB “fit” the function using smoothing splines. To determine whether a unit was significantly modulated during movement, a mean and SD baseline firing rate was taken within the period –4 to –2 s from reach onset. If the mean firing rate in the period from –350 to –850 ms relative to reach onset differed from the baseline mean by >1.25 baseline SDs, the unit was categorized as a reach-modulated unit.

To characterize low-frequency spiking activity, we generated histograms of the LFP phases at which each spike occurred for a single unit to a single LFP channel filtered in the 1–4 Hz band in a 1 s window around movement (–250 ms before to 750 ms after movement onset) across all trials of a session (Fig. 3). For learning comparisons, all units were compared with the same selected M1 and DLS LFP channel on days 1 and 5. These histograms were generated for each unit–LFP channel pair both within and across regions. For every pair, we then calculated the Rayleigh’s *z*-statistic for circular nonuniformity. These *z*-statistics were then used to calculate the percentage of significantly nonuniform distributions across unit–LFP pairs with a significance threshold of *p* = 0.05 (Fig. 3). A significantly nonuniform distribution signifies phase preference for spikes of a unit to an LFP signal. This process was also performed to compare the successful and unsuccessful trials of day 5 (Fig. 3*C*).

#### Single trial to template correlation

Spikes from –4 to 4 s around pellet touch were binned at 20 ms, smoothed with a Gaussian kernel with a SD of 60 ms, and then *z*-scored. Binned, smoothed, and standardized spike counts within the period of –1 to 1.25 s for all units of a single trial were then concatenated into one long vector. The correlation (measured using Pearson’s *r*) between each concatenated single-trial neural activity and the mean template (mean of all trials) was computed, and the mean correlation for each session was reported (Fig. 4).

#### Gaussian-process factor analysis neural trajectory analyses

To characterize single-trial representations of population spiking activity, we used Gaussian-process factor analysis (GPFA; Yu et al., 2009; Lemke et al., 2019) to find low-dimensional neural trajectories, which consisted of the first two factors, for each trial. GPFA analyses were conducted using the MATLAB-based graphical user interface DataHigh (version 1.2; Cowley et al., 2013), 10 ms time bins, and a dimensionality of 5. The mean of all trajectories from successful trials was used as a template, except for the trajectory of the trial being compared. We determined the correlation for each individual trial trajectory with the template by taking the linear correlation between the trajectory of each trial and the mean trajectory across all trials (Fig. 5*B*,*C*; computed in each dimension independently). To compare successful trials, the template was recalculated with the trial removed from the pool, and hence the trial compared was not used to create the template. This was performed specifically for the period between 250 ms before movement onset and 250 ms after retract onset. Since this duration varied across trials, we interpolated each trial such that every epoch (reach onset to touch and touch to retract onset) of each trial was the same length and then calculated the average deviation.

#### Statistical analysis

The linear mixed-effects model (implemented using MATLAB “fitlme”) was used in this study. Using these models accounts for the fact that units, channels, or trials from the same animal are more correlated than those from different animals; thus, it is more stringent than computing statistical significance over all units, channels, or trials (Aarts et al., 2014; Lemke et al., 2019). We fitted random intercepts for each rat and reported the *p* values for the regression coefficients associated with successful or unsuccessful outcome, early (that constituted days 1 and 2) or late (that constituted days 4 and 5) learning, or training session. Linear mixed-effects models were used for testing significance in Figures 1, *D* and *E*, 2*C–E*, 4, *B* and *D*, and 5*C*. Two-sample Kolmogorov–Smirnov tests were used to test whether spike-LFP phase-locking values on days 1 and 5 came from the same distribution (Fig. 3*C*). All statistical analyses were implemented within MATLAB. We fitted random intercepts for each rat and reported the *p* values for the regression coefficients associated with successful or unsuccessful outcome, early or late learning, or training session.

### Data availability

The datasets generated and analyzed in the current study are available from the corresponding author on reasonable request.

## Results

We trained 20 rats on the Whishaw forelimb reach-to-grasp task (Whishaw and Pellis, 1990; Wong et al., 2015) in our in-house-built automated training box that is compatible with electrophysiology (Fig. 1*A*; Gulati et al., 2015; Wong et al., 2015; Ramanathan et al., 2018). We chose this task because of its similarity to skilled learning tasks in humans (Iwaniuk and Whishaw, 2000; Klein et al., 2012) as well as extensive evidence that this task is associated with multiple levels of neural plasticity in the M1 and the cerebellum. Examples of this include changes in long-term potentiation (Rioult-Pedotti et al., 2000), dendritic spine growth (Fu et al., 2012), motor map plasticity in the M1 (Kleim et al., 1998), as well as patterned spiking in the cerebellar cortex (Heck et al., 2002), and, more recently, it has also been demonstrated that cerebellar associative learning underlies reach adaptation (Calame et al., 2021). Importantly, patients with neurologic injury in either region show impairment in this skilled reaching behavior (Zackowski et al., 2002; Sathian et al., 2011). In a subset of rats (*n *=* *10) that were monitored during reach-to-grasp motor skill consolidation, we also recorded neural signals, including single-unit activity and LFPs in M1 and cerebellum (Fig. 2*A*). For the electrophysiology experiments, microelectrodes were implanted (microwire arrays in M1 and tetrodes/polytrodes in cerebellum; see Materials and Methods; Table 1). In the animals that were recorded, training began 5 d after electrode placement surgery.

### Measurement of skilled motor performance

As in other studies that use the Whishaw forelimb reach-to-grasp task, we assessed motor skill learning across two dimensions: speed and accuracy (Fig. 1*B–E*; Ramanathan et al., 2015; Lemke et al., 2019). Accuracy was measured as the percentage of success in retrieving the pellet, and speed was assessed using the time the animal took to perform the full reach-grasp-retract motor sequence. Training lasted for 5 d in automated behavioral boxes (Wong et al., 2015; Ramanathan et al., 2018), and animals performed 100–160 trials each day. Animals were split into the following two categories: experts that achieved a success rate >30% on late days, and nonexperts that did not. On average, success rates of experts increased from 24.0 ± 4.3% to 49.3 ± 3.0% from early to late days (mean ± SEM; mixed-effects model: *p *=* *2.46 × 10^−20^), and reach duration came down from 346.7 ± 71.1 ms on early days to 312.0 ± 63.4 ms on late days (mean ± SEM; mixed-effects model, *p *=* *1.54 × 10^−3^). On average, the success rates of nonexperts increased from 9.5 ± 2.8% to 16.0 ± 3.7% from early to late days (mean ± SEM; mixed-effects model, *p *=* *1.18 × 10^−4^), and reach duration nonsignificantly increased from 714.3 ± 193.2 ms on early days to 857.6 ± 202.6 ms on late days (mean ± SEM; mixed-effects model, *p *=* *0.444; Fig. 1).

### Coordinated movement-related activity emerges across M1 and cerebellum during skill learning

We next evaluated the cerebellum in search of transient LFO dynamics similar to those that were recently shown to emerge in the M1 (Ramanathan et al., 2018; Lemke et al., 2019) while learning this skill. We observed that coordinated LFO (1–4 Hz) activity appeared in LFP signals during movement across M1 and cerebellum in experts (Fig. 2*B*,*C*), but not in nonexperts (Fig. 2*D*). The movement-related LFO power increased from early to late days in both M1 and cerebellum for experts (Fig. 2*D*; M1 baseline-normalized power: early days, 0.56 ± 0.13; late days, 0.72 ± 0.14; mixed-effects model: *t*_(2270)_ = 0.4, *p *=* *9.2 × 10^−6^; cerebellum power, 0.44 ± 0.10 to 0.72 ± 0.24; mixed-effects model: *t*_(1726)_ = 4.8, *p *=* *1.1 × 10^−6^). In nonexperts, M1 LFO power did not change and cerebellar power in LFO power decreased (M1 baseline-normalized power: early days, 0.62 ± 0.08; late days, 0.66 ± 0.12; mixed-effects model, *p *=* *0.614; cerebellum power, 1.71 ± 0.66 to 0.74 ± 0.24, mixed-effects model, *p *=* *2.1 × 10^−11^).

We also analyzed movement-related low-frequency LFP coherence between M1 and cerebellum LFPs, and we found that this also increased only in experts (Fig. 2*E*; early days, 0.20 ± 0.02 coherence; late days, 0.22 ± 0.01 coherence; mixed-effects model: *t*_(7174)_ = 10.0, *p *=* *1.3 × 10^−23^). These increases in LFP power and coherence were not solely a by-product of faster and more consistent movements, since high-LFP power and coherence were not present for fast trials early in training, which we checked in a subset of expert animals. On the contrary, low-frequency LFP coherence between M1 and cerebellum LFPs decreased in the nonexperts (early days, 0.26 ± 0.02 coherence; late days, 0.18 ± 0.01 coherence; mixed-effects model, *p *=* *2.1 × 10^−194^). Since we did not find coemergent LFO activity in nonexperts, our subsequent analyses that looked at spike–LFP phase relations focused only on experts as nonexperts did not show an LFO power increase.

With training, we evaluated reaching submovements around MO, PC, and RO, and whether they became precisely phase locked to 1–4 Hz LFP signals in M1 and cerebellum. We found that all three submovements became precisely phase locked to 1–4 Hz LFP signals in M1 and cerebellum in animals that gained expertise, consistent with what we would expect if this activity was involved in generating submovements within this task (Fig. 2*F*; significant increase in ITC of the M1 LFP locked to MO: mixed-effects model: *t*_(486)_ = 3.9, *p *=* *1.07 × 10^−4^; PC (right at the time of grasp initiation): mixed-effects model: *t*_(486)_ = 8.2, *p *=* *2.7 × 10^−15^; and RO: mixed-effects model: *t*_(486)_ = 11.0, *p *=* *2.0 × 10^−25^; cerebellum LFP locked to movement onset: mixed-effects model: *t*_(818)_ = 3.2, *p *=* *0.0014; pellet touch: mixed-effects model: *t*_(818)_ = 8.0, *p *=* *5.3 × 10^−15^; retract onset: mixed-effects model: *t*_(818)_ = 8.7, *p *=* *1.9 × 10^−17^).

### Coordinated spiking activity emerges across M1 and cerebellum during skill learning

The emergence of coordinated low-frequency activity across M1 and cerebellum was also clearly observed in movement-related spiking activity across M1 and cerebellum. We quantified phase locking of movement-related M1 and cerebellar spikes to 1–4 Hz LFP signals in each region by generating polar histograms of the LFP phase at which each spike occurred for a single unit and LFP channel (Fig. 3*A*). The nonuniformity of the distribution of phases (indicating phase locking) was quantified using a Raleigh test of circular nonuniformity. We compared all task-related M1 and cerebellar units on days 1 and 5 to a representative LFP channel in M1 and cerebellum, and observed an increase in the percentage of M1 and cerebellum units phase locked to both M1 and cerebellum LFP signals with training (Fig. 3*B*, the black vertical dashed lines correspond to the *p *=* *0.05 significance threshold of the natural log of the *z* statistic; M1 unit–M1 LFP pairs: 35.2% day 1 to 55.4% day 5, *p *=* *5.3  × 10^−9^, Kolmogorov–Smirnov test; M1 unit–cerebellum LFP pairs: 40.8–70.8%, *p *=* *1.6 × 10^−15^, Kolmogorov–Smirnov test; cerebellum unit–M1 LFP pairs: 87.5–82.2%, *p *=* *0.12, Kolmogorov–Smirnov test; cerebellum unit–cerebellum LFP pairs: 63.1–73.9%, *p *=* *1.1 × 10^−4^, Kolmogorov–Smirnov test). All the pairs showed a significantly increased phase locking, except cerebellum unit–M1 LFP pairs, where a high proportion of units were phase locked to M1 even during early days. These results suggest that coordinated low-frequency activity emerges across M1 and cerebellum during skill learning. However, cerebellar units are extensively phase locked to M1 LFOs E in training.

Next, we also explored these relations for successful and unsuccessful trials on day 5. We found that all four pairs showed significant phase locking of M1 and cerebellar units to 1–4 Hz M1 and cerebellum LFPs for successful trials (Fig. 3*C*, the black vertical dashed lines correspond to the *p *=* *0.05 significance threshold of the natural log of the *z*-statistic; M1 unit – M1 LFP pairs: 30.1% for unsuccessful trials vs 51.3% for successful trials, *p *=* *4.0 × 10^−4^, Kolmogorov–Smirnov test; M1 unit – cerebellum LFP pairs: 46.4–70.0%, *p *=* *6.2 × 10^−8^, Kolmogorov–Smirnov test; cerebellum unit – M1 LFP pairs: 51.1–82.2%, *p *=* *2.7 × 10^−7^, Kolmogorov–Smirnov test; cerebellum unit – cerebellum LFP pairs: 62.5–67.0%, *p *=* *9.5 × 10^−5^, Kolmogorov–Smirnov test).

### Reorganization of neural dynamics in M1 and cerebellum with skill learning

We also investigated the consistency of single-trial population spiking activity by computing the correlations between single-trial neural activity and the trial-averaged template across all units in a session (Fig. 4). In early sessions, trial-to-trial neural firing was more inconsistent compared with later sessions, while later sessions were consistently associated with a stereotyped sequence of unit activations that also matched PETHs. This was observed in both M1 (Fig. 4*A*) and cerebellar (Fig. 4*C*) activity. Across the sessions from all rats, we observed a significant increase in template correlation among trials (Fig. 4*B*,*D*; linear mixed-effects model: M1: *t*_(2445)_ = 10.3, *p *=* *3.5 × 10^−24^; cerebellum: *t*_(2421)_ = 6.4, *p *=* *2.1 × 10^−10^), indicating that trial-to-trial variability in M1 and cerebellum neural activity were reduced with skill consolidation.

### Skilled movement representation in M1 and cerebellum

Last, we explored the representation of successful and failed reaches in M1 and cerebellum. We used GPFA to find low-dimensional neural trajectory representations of population spiking activity in M1 and cerebellum on individual trials (Yu et al., 2009; Lemke et al., 2019; Fig. 5*A*) and then compared trajectories for successful and unsuccessful trials in early and late learning. We observed a difference between trajectories for successful and unsuccessful trials in M1 and cerebellum. To compare successful and unsuccessful trials, we computed the correlation between the mean neural trajectory for successful trials, that is, the “successful template,” and the neural trajectory of each individual trial (Fig. 5*B*) during the period from 250 ms before movement onset to 250 ms after retract onset (Fig. 5*C*). This period encompassed the movement onset and pellet contact for grasping and retraction of the forelimb. Since trials differed in the duration of this period, we interpolated trajectories such that they were all the same length. Neural trajectories for unsuccessful trials had significantly lower correlation than successful trials for M1, but not for cerebellum (Fig. 5*C*; E M1, *p *=* *3.5 × 10^−6^; E cerebellum, *p *=* *0.144; L M1, *p *= 3.6 × 10^−5^; L cerebellum, *p *=* *0.121 mixed-effects model with Bonferroni correction for multiple comparisons). This indicates that spiking activity during the reach-to-grasp task is not remarkably different for successful versus unsuccessful trials in the cerebellum.

## Discussion

In summary, we found that coordinated low-frequency activity emerged across M1 and cerebellum, which was linked to the emergence of faster and more accurate reaching movements. We found interindividual variability within animals, and we found that LFO activity emerged in animals that were able to gain expertise in the task within 5 d. Previous reports indicate that slow improvements in accuracy can continue to occur with extended practice in the rodent reach-to-grasp task (Lemke et al., 2019) and that the behavioral exploration phase can vary between animals when the target location is switched in this reaching task (Kondapavulur et al., 2022). Our paradigm involved training for a contiguous 5 d, and we observed emergent LFO trends in animals that gained proficiency in the task. We further found that coordinated spiking activity in both these regions was linked to accurate reach-to-grasp movements. Our work details the mesoscopic transmission in cortico-cerebellar networks and how it evolves with expert skill learning as well as how skilled reaching has a motor cortical and cerebellar cortical reliance and spiking activity differences in these regions for successful behavior.

### Role of M1 and cerebellum in motor skill learning and execution

M1 has a well established role in motor learning as well as movement execution (Peters et al., 2017). In particular, M1 is critical for the execution of skilled dexterous movement (Iwaniuk and Whishaw, 2000; Guo et al., 2015; Peters et al., 2017; Lemke et al., 2019). Our work is consistent with this notion as we also see that M1 activity patterns are different for successful pellet retrieval (Figs. 3*C*, 4*A*, 5). The projection of M1 to the cerebellum is thought to mediate fine-tuning of the movement. Cerebellar neurons in the cortex and in the deep nuclei are known to be modulated around several movement events. Perturbation of M1 input to cerebellum or direct manipulation of cerebellum itself is shown to delay movement initiation and to increase movement variability and duration (Conrad and Brooks, 1974; Becker and Person, 2019; Nashef et al., 2019; Guo et al., 2021). Our work is also consistent with these observations as we found that precise, accurate movements had more consistent cerebellar spiking–M1 LFP LFO coordination for successful reaches on day 5 in expert animals (Fig. 3*C*). However, we found that GPFA-derived neural trajectories were more correlated for successful trials in M1 only (Fig. 5). This indicates that while cerebellum spike–M1 LFP coordination (with latter being aggregate synaptic input within an area) was higher on later phases of training for successful movements, just the spiking activity in cerebellar cortex was not different for successful versus failed reaches. In addition to this role in increasing movement precision, cerebellar cortex is also theorized to contribute to task-relevant dimensionality expansion that can aid in flexible computation and enhance learning (Marr, 1969; Albus, 1971; Litwin-Kumar et al., 2017). This notion of dimensionality expansion was confirmed experimentally with the observations of high correlations among granule cell activity when mice expertly exerted pushing control over a manipulandum in a forelimb movement task (Wagner et al., 2019). This work also showed an increase in emergent shared variance in M1 and cerebellar cells. Our increased M1–cerebellum LFP coherence with skill learning is consistent with this observation. Neural network models of cortico-cerebellar networks show that cerebellar feedback improves the rate of learning and that the cerebellar network also carries task representation (Boven et al., 2023). Our experimental data support this notion as well. We observed that M1–cerebellum LFP coherence increased with learning, and we observed movement-modulated units in the cerebellum. One of our observations also showed that M1 LFP–cerebellar units showed strong coordination in the low-frequency range early on in training (Fig. 3*B*). This might suggest that cerebellar activity was critical during reach-to-grasp skill acquisition and is consistent with the notions of M1 being input driven, and is also consistent with the cerebellar contributions to the acquisition of skilled volitional movements (Bloedel et al., 1997; Sauerbrei et al., 2020).

### Coordinated oscillatory dynamics across motor networks

One of our key findings here is on low-frequency activity across M1 and cerebellum as an important marker of skilled motor control. We found evidence of such activity at the level of neural spiking and LFPs during the performance of dexterous task in rats. It is noteworthy that similar LFOs were recently shown to be disrupted in M1 poststroke and tracked recovery (Ramanathan et al., 2018). This work also boosted M1 LFOs through electric stimulation to promote recovery. Recently, there has also been an interest in cerebellar stimulation for stroke recovery (Machado et al., 2009; Shah et al., 2017; Abbasi et al., 2021), but a biomarker in cortico-cerebellar networks that can be a target for closed-loop electric stimulation for stroke recovery is lacking. Future work can test whether the LFOs we found in cortico-cerebellar networks of healthy animals with skill consolidation here can also serve as a biomarker for motor function during recovery from stroke. Mesoscopic biomarkers such as LFPs present a lower translational barrier in clinical populations.

Cortical LFOs can be used to decode reach-related activity and predict spiking phase across multiple behavioral states (Mollazadeh et al., 2009; Hall et al., 2014b). Such activity is also correlated with multiphasic muscle activations and timing of movements (Stefanics et al., 2010; Churchland et al., 2012; Flint et al., 2012; Hall et al., 2014b). Recent work also suggests that oscillatory dynamics reflect an underlying dynamical system (Churchland et al., 2012). This previous work argues that LFOs represent an intrinsic property of motor circuits associated with precise movement control. Our findings extend this body of work by showing LFO dynamics in both M1 and cerebellum (Fig. 2). The exact origin of LFOs and underlying generators remains unknown. While reach-related LFOs may have involved striatum (Lemke et al., 2019) or thalamocortical activity (Dossi et al., 1992), so far, our results here raise the possibility of cerebellar involvement. Further work can probe interactions between M1 and the broader motor network to pinpoint the drivers of the electrophysiologic changes seen during skill learning.

## Synthesis

Reviewing Editor: Silvia Pagliardini, University of Alberta Decisions are customarily a result of the Reviewing Editor and the peer reviewers coming together and discussing their recommendations until a consensus is reached. When revisions are invited, a fact-based synthesis statement explaining their decision and outlining what is needed to prepare a revision will be listed below. The following reviewer(s) agreed to reveal their identity: NONE. Note: If this manuscript was transferred from JNeurosci and a decision was made to accept the manuscript without peer review, a brief statement to this effect will instead be what is listed below.

This manuscript presents some novel observations during motor learning, opening the door to study of M1-cerebellum dynamics. The authors found peri-movement low frequency oscillations in cerebellum that had previously seen in M1. They found that M1

and cerebellum LFO are coherent and that coherence increases with improved performance over 5 days. They found that spiking in each brain area is phase locked to this shared LFO (although interestingly cerebellar units do not show locking to cerebellar

LFO as well as they do to M1 LFO).

The authors addressed comments and concerns expressed by reviewers in the previous submission.

Without lead-lag differences, I am curious why cerebellar units follow M1 LFP more than cerebellar LFP. I’d explore that in future work using lead-lag analyses of lfp-lfp and lfp-spike.
